# The circulatory levels and bone expression of MIR21, 34a, 155 and their target genes in a section of Egyptian Population

**DOI:** 10.1038/s41598-024-77643-9

**Published:** 2024-11-13

**Authors:** Rasha A El-Tahan, Ghaleb A Oriquat, Islam Sorour, Sherif M Salem, Maher A Kamel, Trez N Michel, Nehal Abu-Samra

**Affiliations:** 1 Department of Biochemistry, Medical Research Institute, Alexandria University, Alexandria, 21561 Egypt; 2 Department of Medical Laboratory, Amman University, Faculty of Allied Medical Sciences, Amman, 19328 Jordan; 3 Department of Neurosurgery, Faculty of Medicine, Alexandria University, Alexandria, 21131 Egypt; 4 Research Projects Unit, Pharos University in Alexandria, Alexandria, 21648 Egypt; 5 Department of Physiology, Medical Research Institute, Alexandria University, Alexandria, 21561 Egypt; 6 Department of Basic Sciences, Faculty of Physical Therapy, Pharos University in Alexandria, Alexandria, 21648 Egypt

**Keywords:** Bone quality, MicroRNA, Bone metabolism, Osteocalcin, Egyptian population, Biochemistry, Molecular biology, Neuroscience

## Abstract

Bone tissue is constantly regenerated and repaired through a finely balanced process known as bone remodeling. Many miRNAs act as regulators of the signaling pathways involved in bone metabolic processes to maintain tissue homeostasis. This study aimed to assess the circulating levels of MIR21, MIR34a, and MIR155 in human serum and their bone expression, and the expression of bone turnover-related genes which can reflect the bone quality. This prospective study was conducted on 60 patients (30 males and 30 females) indicated for surgical interventions for neural decompression +/- fixation. Relative quantification of expression of MIR21, miR34a, and MIR155 and bone related genes was assayed using PCR. The serum levels of osteocalcin and Serum Bone Alkaline Phosphatase (sBAP) were assayed using a human ELISA kit. The main finding of the present work was the strong positive association between the circulating levels of only miR21 and MIR155 and their bone expression in the population under study and with bone markers and target genes Also, a positive association was found between both bone expression and circulating MIR21 levels with age and sBAP. These results suggest that the circulating levels of these microRNAs as early markers for the predication of bone quality.

## Introduction

Bone tissue is a major structural component of the skeleton. It is a key player in vital functions in human physiology including fortification, movement, and support of vital body organs, mineral storage, homeostasis, and the housing of multiple progenitor cells, such as hematopoietic mesenchymal cells^1^. It is constantly regenerated and repaired through a finely balanced process known as bone remodeling which involves the elimination of aged or damaged bone by osteoclasts^2,3^. Over time, the material properties of bone change, resulting in a decline in bone quality and an increased risk of fractures, especially in elderly people and postmenopausal women^4^. The ability to predict fracture risk, and monitor bone healing and regeneration is critical. The current gold standard for bone quality assessment is the measurement of bone mineral density (BMD) by dual-energy X-ray absorptiometry (DXA)^5^. However, BMD only provides information on bone quantity and provides no information on bone microstructure and changes in bone mass may take years to be reflected in a change in fracture rate^6^. So, there is a clinical need for the development of novel methods and technologies for early detection and monitoring of the changes in bone quality, to allow for the implementation of effective and timely preventative strategies. In pursuit of specific circulating markers of bone strength, the field of micro-ribonucleic acids (miRNAs) and bone is currently the focus of research.

MicroRNAs (miRNAs) are small non-coding RNAs that act as a negative regulator of gene expression post-transcriptionally and are critical for development, cell differentiation, and maturity^7^. Due to the stability of miRNAs and the sensitive quantitative analysis, the specific miRNAs of bone quality will become the future method of identifying those at risk of osteoporosis and predicting fracture. Also, expression profiling allows the concurrent measurement of different molecular markers of bone turnover-related genes, and miRNAs can facilitate a more comprehensive assessment of bone health.

The signaling pathways involved in various bone metabolic processes to maintain tissue homeostasis include; transforming growth factor-β (TGFβ), bone morphogenetic protein receptor type 2 (BMPR2), TGFβ-responsive and receptor activator of nuclear factor-κB ligand (RANKL); also known as TNF superfamily member 11 (TNFSF11), and matrix metalloproteinase (MMP) signaling pathways. Bone metabolism is controlled by osteoblasts, which promote bone formation, and osteoclasts, which promote bone absorption^8^. Bone morphogenetic proteins (BMPs) belong to the transforming growth factor (TGFβ) superfamily and are essential for terminal-osteoblast differentiation and promoting bone formation. The effects of BMP2 are predominantly mediated through BMPR2 resulting in activation of receptor-regulated SMADs (R-SMADs), SMAD1, 5, or 8, which interacts with SMAD4, enters the nucleus, and activates transcription. SMAD7 is an inhibitory SMAD that inhibits SMAD1/5/8 activation and functions to negatively regulate BMP signaling. Therefore, the interaction of BMPR2, SMAD1, 5, and 7 functions in a manner that is crucial in controlling the bone anabolic response. The BMPR2 gene is thought to be the key gene that controls bone formation^9,10^. The TNFSF11 (RANKL), recognized by its specific receptor called TNF receptor superfamily member 11a (TNFRSF11A) or RANK present at the surface of bone marrow monocytes/macrophages to induce osteoclastogenesis and gives signals to produce MMP9 and bone resorption^11,12^. All of these signaling pathways were found to be directly or indirectly regulated by miRNAs^13–22^.

The investigation of specific miRNA in bone tissues and secretions could evolve as a novel indirect estimation of bone quality status in both men and pre/postmenopausal women. So, the present study focused on three miRNAs (MIR21, MIR34a, and MIR155) that are paneled to be markers of bone quality in human subjects. Thus, the major objective of this study was to assess the extent to which assessment of circulating levels of the selected miRNAs in human serum can reflect their bone expression, expression of bone turnover-related genes, and bone quality. Addressing these issues will improve our understanding of bone biology and improve methods to assess bone quality, especially in postmenopausal females.

## Results

### Clinical data of the studied population

The study was conducted on 60 patients who underwent spine surgery. Thirty patients were males with a mean age of 48.7 ± 13.5 years. Fifteen patients were premenopausal females with a mean age of 32.5 ± 12.4 years. Another 15 patients were postmenopausal females with a mean age of (55.6 ± 4.9). All patients under study have a normal serum range of bone biomarkers; osteocalcin and sBAP (Table 1).

**Table 1:** Clinical data of the study population.

	Males	Females	
		Premenopausal	Postmenopausal
Age (year)	48.7 ± 13.5	32.5 ± 12.4	55.6 ± 4.9
Weight (kg)	80.7 ± 11.5	72.8 ± 5.4	75.5 ± 9.2
Highet (cm)	174.3 ± 14.1	162.1 ± 12.8	160.51 ± 11.5
BMI (Kg/m^2^)	26.5 ± 2.1	27.7 ± 2.3	29.3 ± 2.8
Osteocalcin (ng/ml)	8.46 ± 3.19	9.24 ± 4.11	11.06 ± 4.85
sBAP (ng/ml)	12.84 ± 4.25	10.61 ± 3.84	14.51 ± 6.77

## Circulatory and bone levels of microRNAs

### Circulatory and bone levels of MIR21

**Fig. 1:**
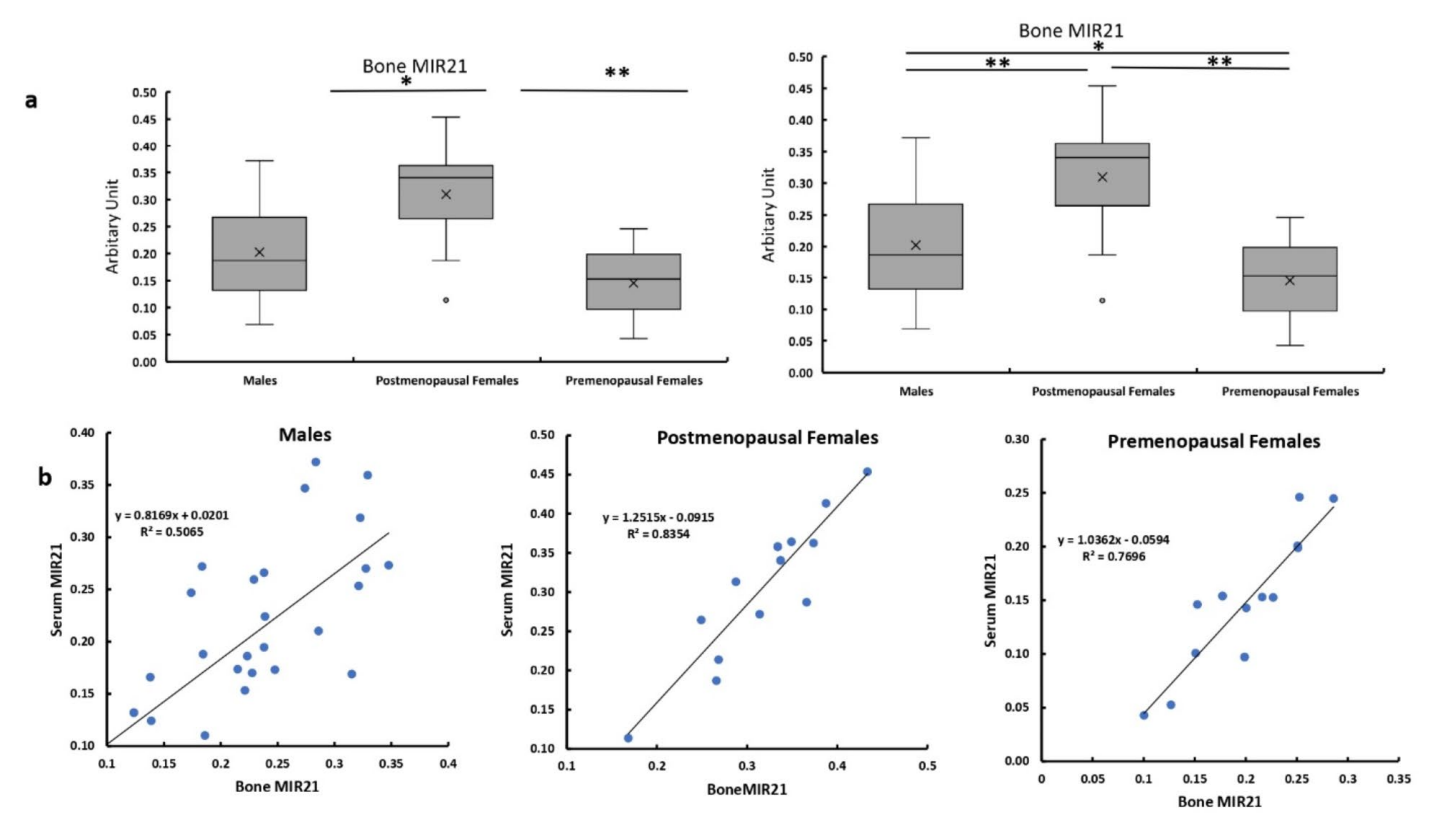
Circulatory and bone levels of MIR21 in all studied groups.** a** Serum MIR21 expression levels in bone and serum in males, and post and premenopausal females.** b** Correlation between MIR21 bone expression with its circulatory levels in males, and post and premenopausal females.

The circulatory level of MIR21 was significantly higher in the postmenopausal females compared with the males and premenopausal females. Also, in bone tissues, the postmenopausal females have significantly higher expression of MIR21 compared with males and premenopausal females. While the premenopausal females have significantly lower bone expression compared with that of males (Fig. 1a).

The level of MIR21 bone expression showed a significant correlation with its circulatory level in males. While it was strongly correlated with circulatory levels in both pre and postmenopausal females (Fig. 1b).

### Circulatory and bone levels of MIR34a

**Fig. 2:**
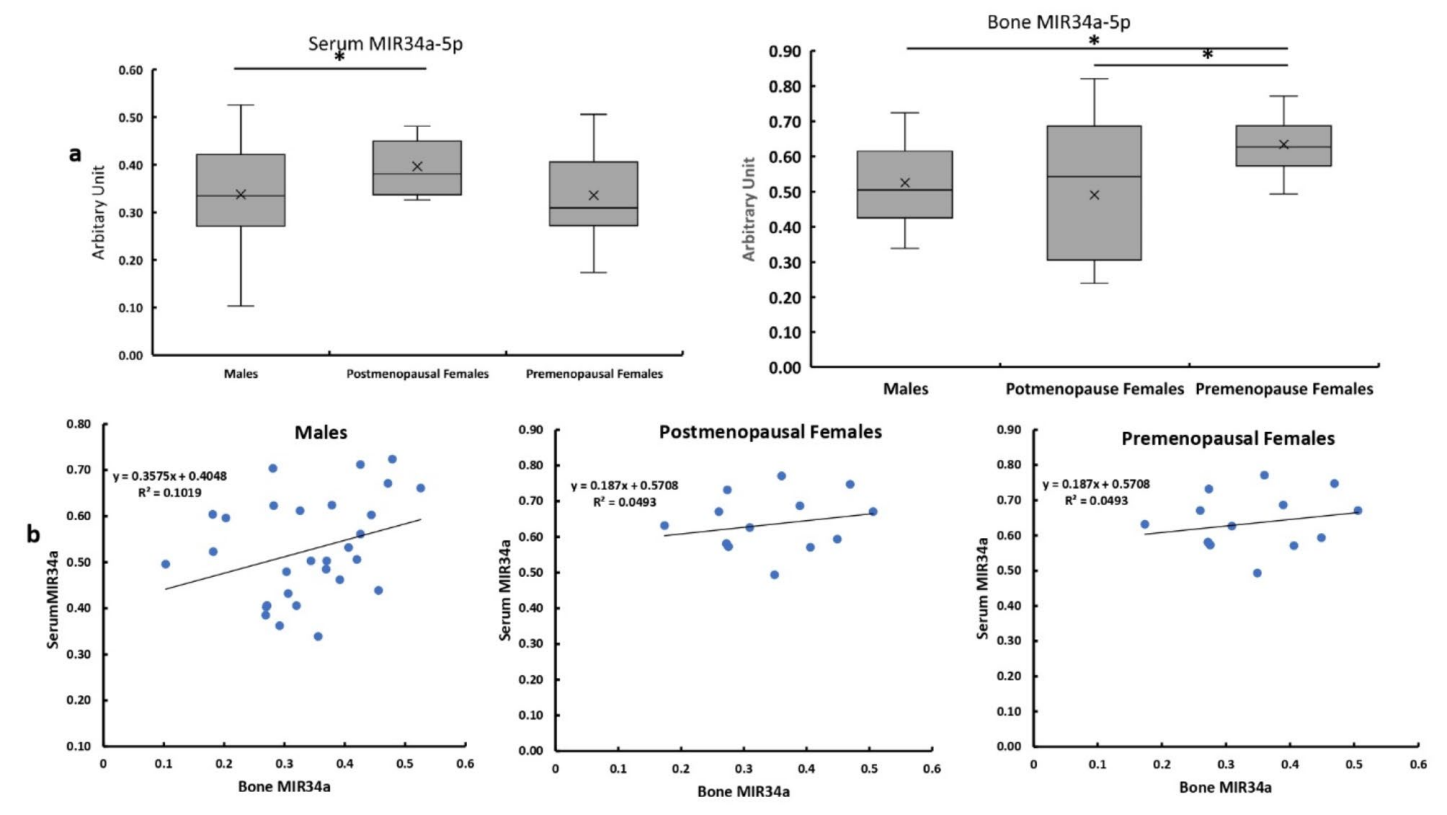
Circulatory and bone levels of MIR34a in all studied groups** a**Serum MIR34a expression levels in bone and serum in males, post and premenopausal females. ** b**Correlation between MIR34a bone expression with its circulatory levels in males, and post and premenopausal females**.**

The circulatory level of MIR34a was slightly but significantly higher in the postmenopausal females compared with the males with no significant difference compared with premenopausal females. In bone tissues, the premenopausal females have significantly higher expression of MIR34a compared with males and postmenopausal females with no significant difference between males and postmenopausal females (Fig. 2a). The level of MIR34a bone expression showed no significant correlation with its circulatory levels in all studied patients (Fig. 2b).

## Circulatory and bone levels of MIR155

**Fig. 3:**
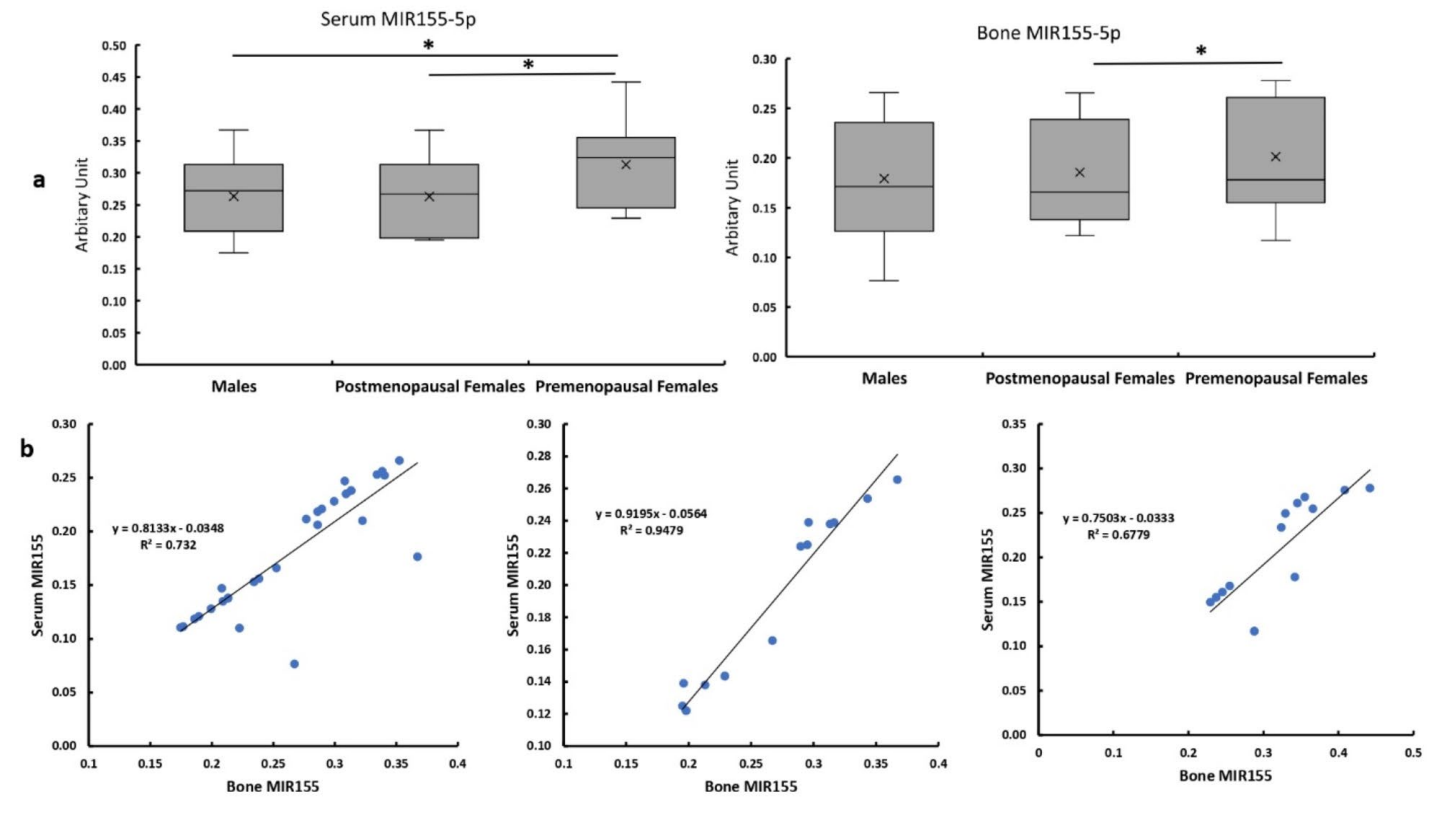
Circulatory and bone levels of MIR155 in all studied groups**.   a **Serum MIR155 expression levels in bone and serum in males, post and premenopausal females. **b **Correlation between MIR155 bone expression with its circulatory levels in males, and post, and premenopausal females**.**

The circulatory level of MIR155 was significantly higher in the premenopausal females compared with the males and postmenopausal females. In bone tissues, premenopausal females have significantly higher expression of MIR155 compared with postmenopausal females. While no significant differences between bone expression of MIR155 in males and both subgroups of females (Fig. 3a). The level of MIR155 bone expression showed a significant strong correlation with its circulatory level in all studied patients (Fig. 3b).

### Bone expression of target genes

#### Bone expression of BMPR2

**Fig. 4:**
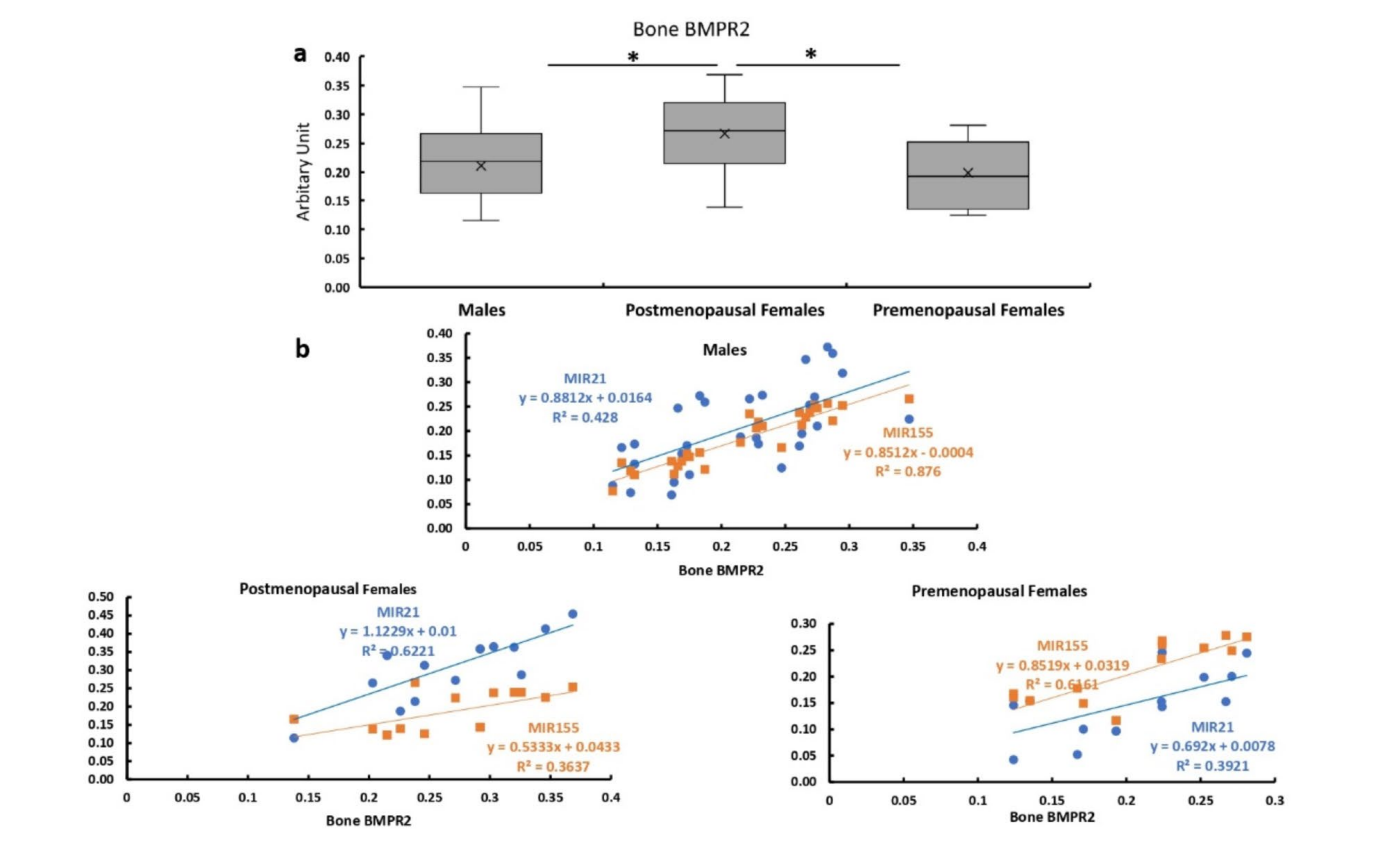
Bone expression of BMPR2 in all studied groups. **a**  Bone expression of BMPR2 in males, and post and premenopausal females. **b** Correlation between bone expression of BMPR2 and expression levels of MIR21 and MIR155 males, and pre and postmenopausal females.

The bone expression of BMPR2 was significantly higher in the postmenopausal females compared with the males and premenopausal females, with no significant difference between males and premenopausal females (Fig. 4a).

The bone expression of BMPR2 showed significant positive correlations with the expression levels of MIR21 and MIR155 in males and both pre and postmenopausal females (Fig. 4b).

### Bone expression of TNFSF11

**Fig. 5:**
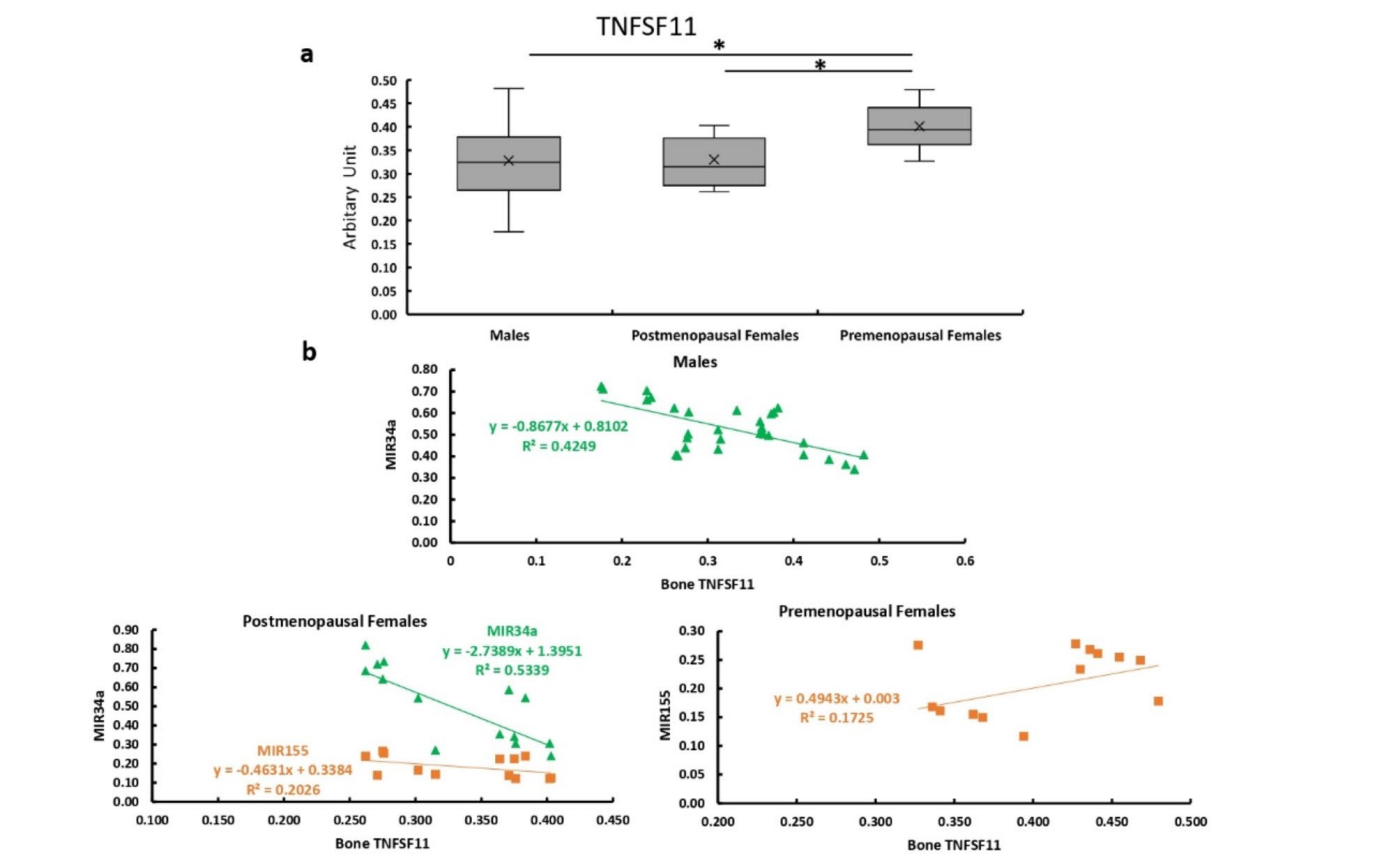
Bone expression of TNFSF11 in all studied groups. **a** Bone expression of TNFSF11 in males, and post and premenopausal females. **b** Correlation between bone expression of TNFSF11 and expression levels of MIR34a and MIR155 in males, and post and premenopausal females

The bone expression of TNFSF11 was significantly higher in the premenopausal females compared with the males and postmenopausal females with no significant difference between males and postmenopausal females (Fig. 5a). The bone expression of TNFSF11 showed significant negative correlations with the expression levels of MIR34a in both males and postmenopausal females. While it showed no significant correlations in premenopausal females and showed a positive correlation with MIR155 in premenopausal females (Fig. 5b).

### Bone expression of SMAD1

**Fig. 6:**
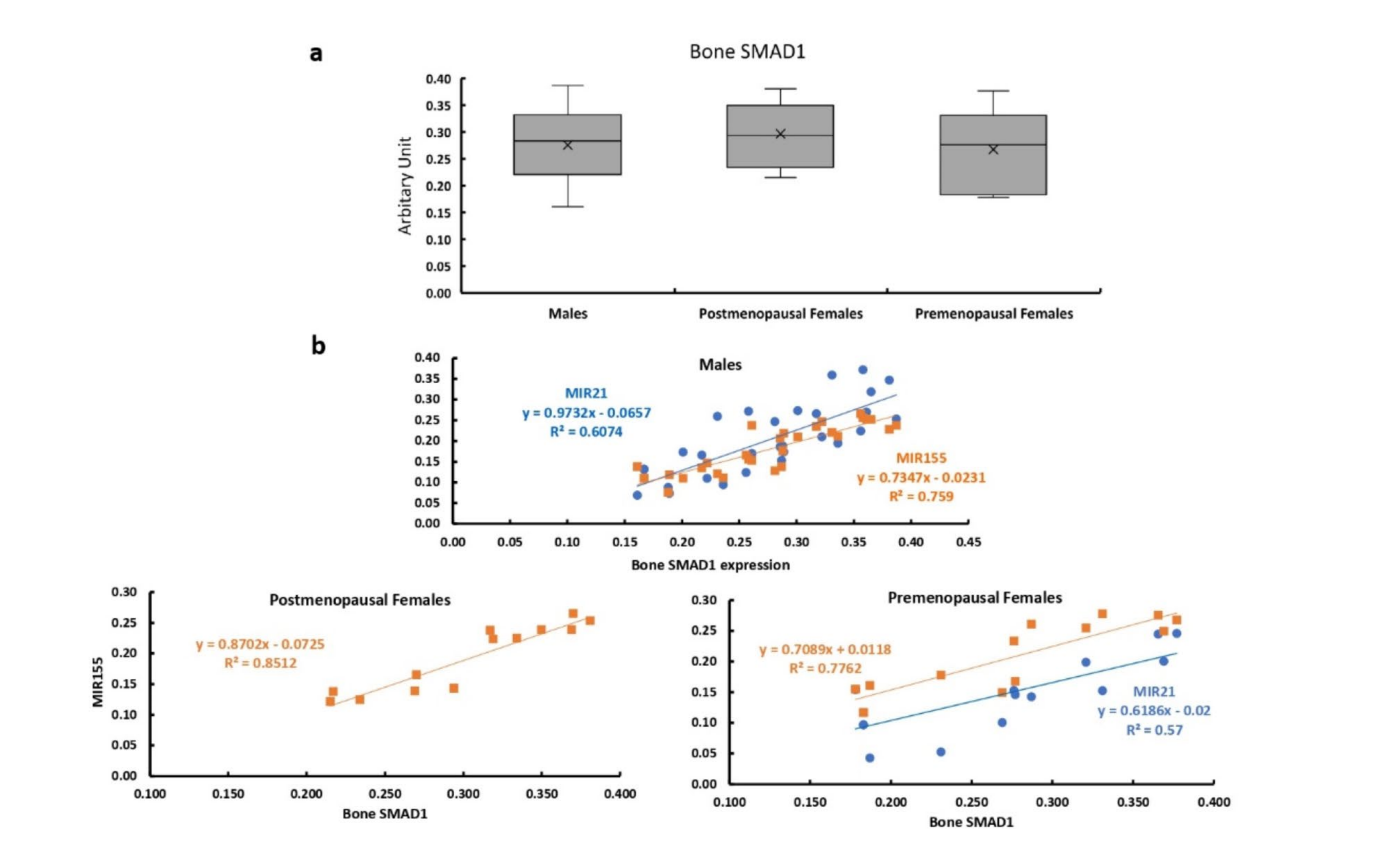
Bone expression of SMAD1 in all studied groups. **a** Bone expression of SMAD1 in males, and post and premenopausal females.  **b** Correlation between bone expression of SMAD1 and expression levels of MIR21 and MIR155 in males, and post and premenopausal females.

The bone expression of SMAD1 showed no significant differences between males and females and between premenopausal and postmenopausal females (Fig. 6a). The bone expression of SMAD1 showed significant positive correlations with the expression levels of MIR155 in all studied patients. While it was significantly positively correlated with the expression level of MIR21 in males and premenopausal females only (Fig. 6b).

### Bone expression of SMAD5

**Fig. 7:**
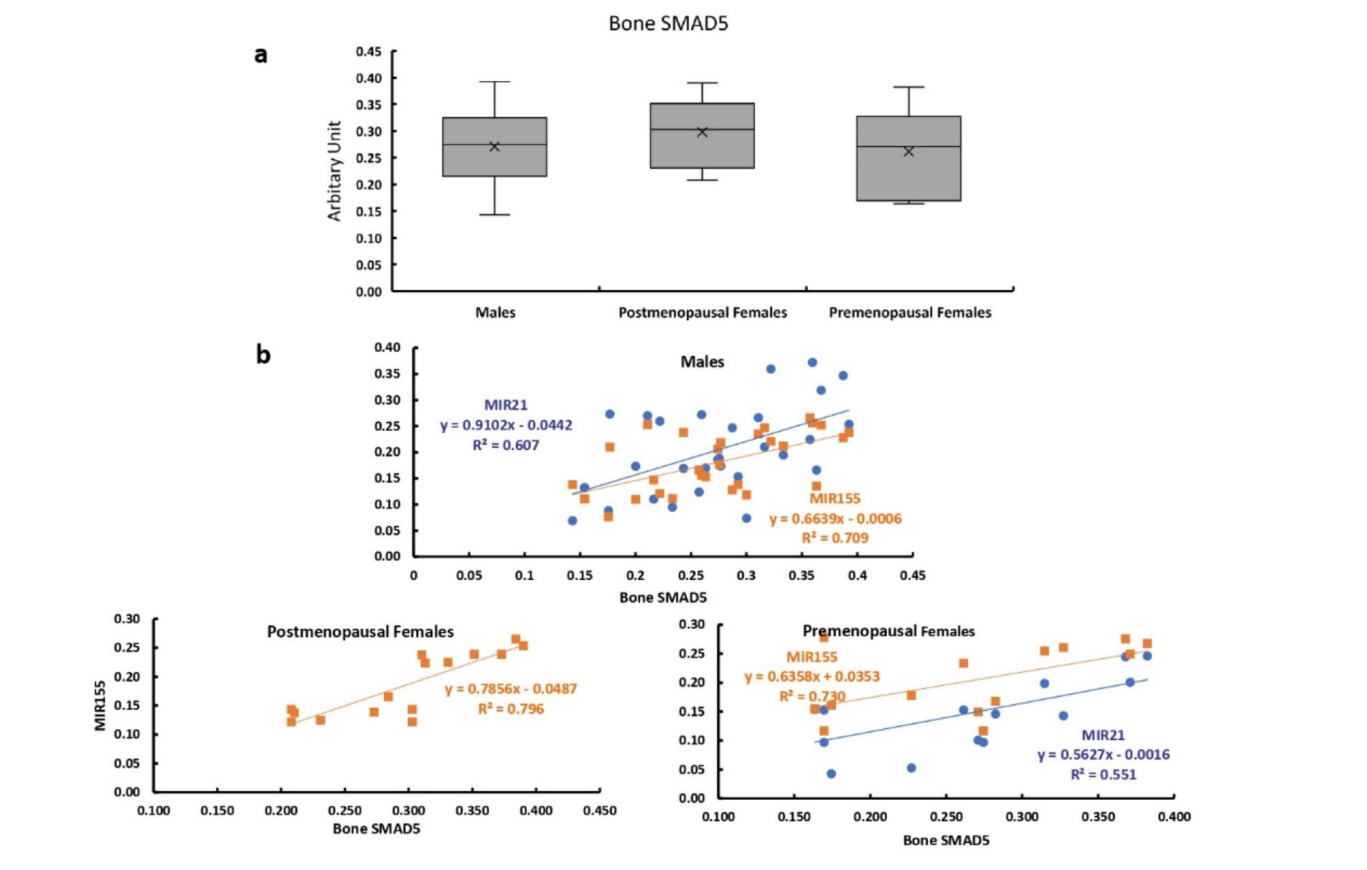
Bone expression of SMAD 5 in all studied groups. **a** Bone expression of SMAD5 in males, and post and premenopausal females.  **b** Correlation between bone expression of SMAD5 and expression levels of MIR21 and MIR155 in males, and post and premenopausal females.

The bone expression of SMAD5 showed no significant differences between males and females and between premenopausal and postmenopausal females (Fig. 7a). The bone expression of SMAD5 showed significant positive correlations with the expression levels of MIR155 in all studied patients. While it was significantly positively correlated with the expression level of MIR21 in males and premenopausal females only (Fig. 7b).

### Bone expression of SMAD7

**Fig. 8:**
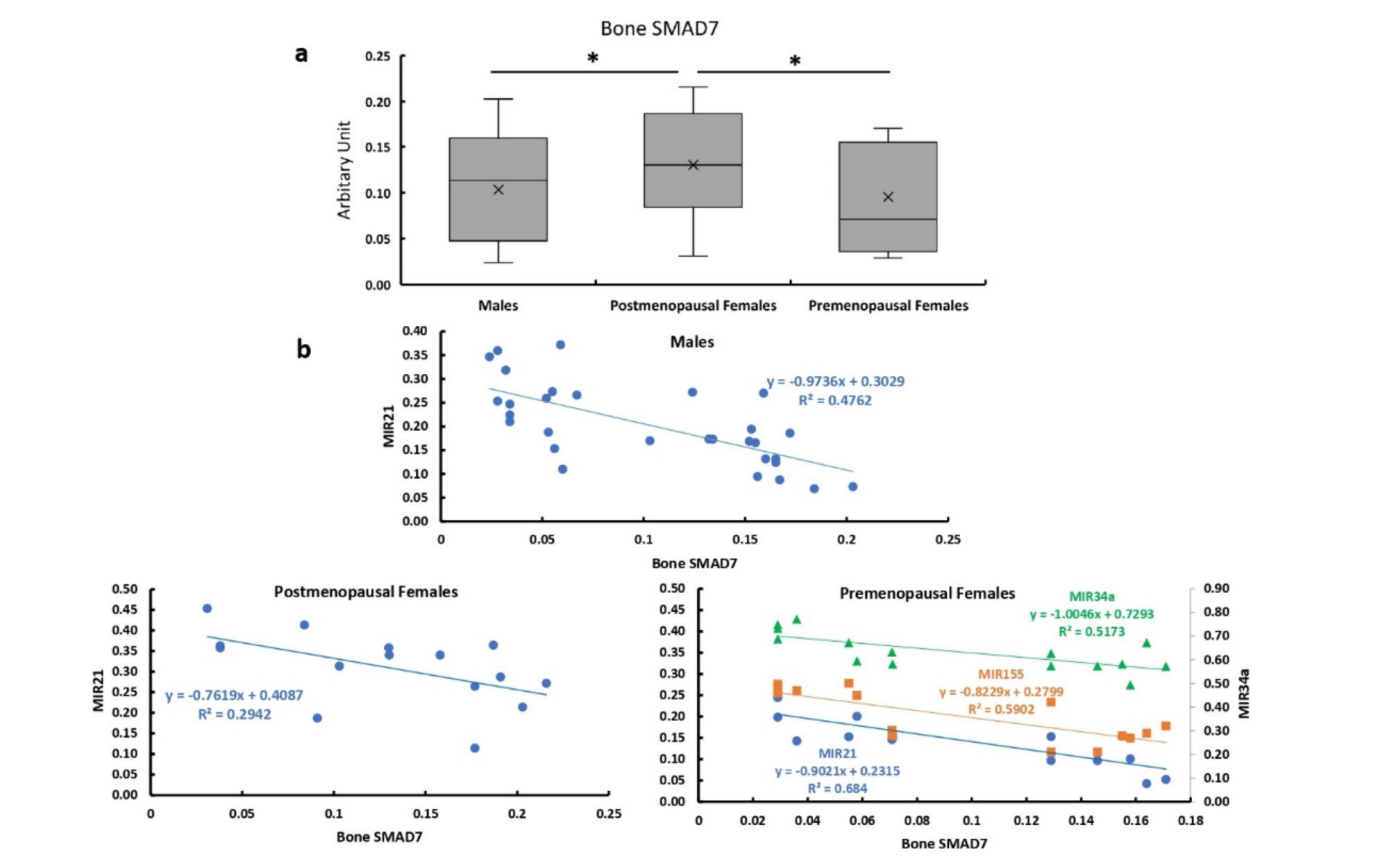
Bone expression of SMAD 7 in all studied groups. **a** Bone expression of SMAD7 in males, and post and premenopausal females.  **b** Correlation between bone expression of SMAD 7 and expression levels of MIR21, MIR34a,  and MIR155 in males, and post and premenopausal females.

The bone expression of SMAD7 was significantly higher in the premenopausal females compared with the males and postmenopausal females with no significant difference between males and postmenopausal females (Fig. 8a). The bone expression of SMAD7 showed significant negative correlations with the expression levels of MIR21 in all studied patients. While it was significantly negatively correlated with the expression level of MIR34a and MIR155 in premenopausal females (Fig. 8b).

### Bone expression of MMP9

**Fig. 9:**
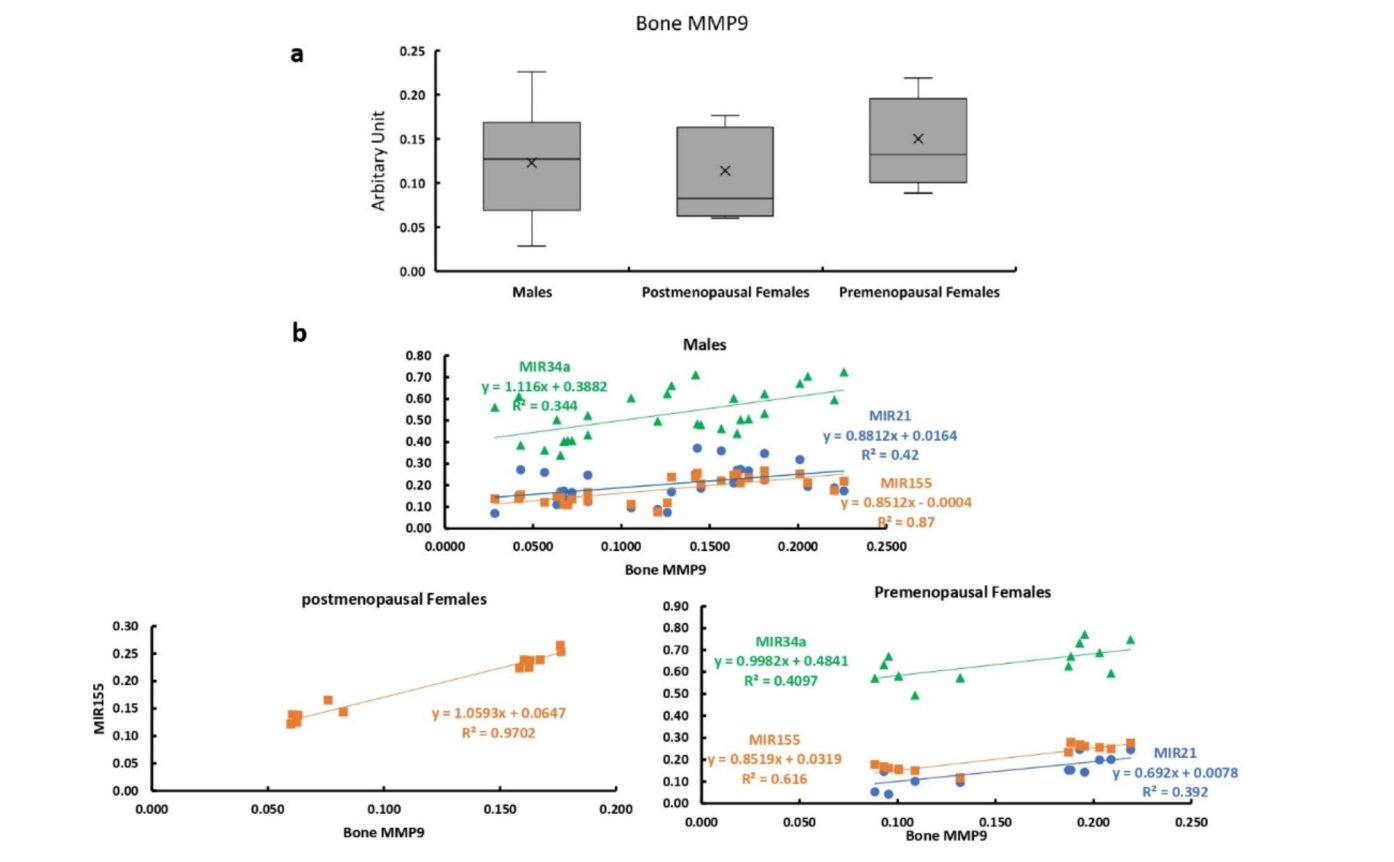
Bone expression of MMP9 in all studied groups. **a** Bone expression of MMP9 in males, and post and premenopausal females.  **b** Correlation between bone expression of MMP9 and expression levels of MIR21, MIR34a, and MIR155 in males, and post and premenopausal females.

The bone expression of MMP9 showed no significant differences between males and females and between premenopausal and postmenopausal females (Fig. 9a). The bone expression of MMP9 showed significant positive correlations with the expression levels of MIR21 and MIR34a, in males and premenopausal females and positively correlated with the bone expression of MIR155 in all studied patients (Fig. 9b).

### Association between the studied miRNAs and age and sBAP

The serum and bone expression of MIR21 were positively associated with age and sBAP in all studied patients. (Table 2). Only the bone expression of miR34a is positively associated with age in all groups. On the other hand, the serum and bone expression of MIR155 are not significantly associated with age and negatively associated with sBAP in all studied patients (Table 2).

**Table 2:** Correlations coefficient between serum (S) and bone (B) levels of microRNAs and age.

			S. MIR21	B. MIR21	S. MIR34a	B. MIR34a	S. MIR155	B. MIR155
Age		Males	0.473^**^	0.441^**^	-0.302	0.464^**^	0.043	-0.219
	**Females**	Premenopausal	0.557^*^	0.721^**^	0.224	0.863^**^	0.369	0.356
		Postmenopausal	0.637^*^	0.501^*^	0.402	0.621^*^	0.431*	0.460*
sBAP		Males	0.441*	0.247	0.241	0.394*	-0.407*	-0.336*
	**Females**	Premenopausal	0.38*	0.331*	0.305	0.221	-0.372	-0.278
		Postmenopausal	0.557*	0.426*	0.229	0.163	-0.435*	-0.422*

*. Correlation is significant.

## Discussion

In pursuit of specific biomarkers of bone health and quality, the field of microRNAs (miRNAs) and bone is currently the focus of research. Thus, the assessment of circulating levels of specific miRNAs and the extent to which their serum can predict their bone expression and the expression of their targets in a well-defined population will be of great importance to finding ideal serum marker(s) of bone health status. So, in this prospective study, we focused on the quantification of three miRNAs; MIR21, MIR34a, and MIR155 in serum and vertebral bone samples from Egyptian men and pre-and postmenopausal women, and correlated these levels with serum bone biomarkers, and the bone expression of genes controlling bone health.

Estimating the bone expression of microRNAs which target genes controlling bone formation and resorption through assessment of their circulatory levels presents a promising method for predicting bone health and diseases. The main finding of the present study is the strong positive association between the circulating levels of both MIR21 and MIR155 and their bone expression levels in the populations under study (males, premenopausal females, and postmenopausal females) which indicates their potential as a candidate serum marker of bone quality. Such an association is not found between the circulatory level and bone expression of MIR34a.

In the present study, we focused on the sex differences in the circulating levels and expression of the studied miRNAs and their roles in supervising the canonical BMP/SMADs signaling pathways in the vertebral bone of the study populations. The circulating levels and bone expression of MIR21 showed its highest values in the postmenopausal females who have significantly higher levels compared with the males and premenopausal females with the lowest levels observed in the premenopausal females. This pattern of changes suggests the possible regulation by the estrogen hormone which plays an important role in the bone remodeling process and affects bone quality. The cell death signal; FasL is a direct target of MIR21^23^, and so the induced expression of MIR21 is associated with osteoclastogenesis^24^. It was demonstrated that estrogen through its receptor α (ERα) directly suppresses the maturation of miRNAs^25^, and reduces MIR21 biogenesis resulting in an increase in FasL protein levels and caspase-3 activity during TNFSF11 (RANKL)-induced osteoclastogenesis and causing the induction of apoptosis of osteoclasts. The action of estrogen was blocked in ERα-deficient cells. Thus, it was concluded that osteoclasts are direct targets of estrogen and the biological effects of estrogen are controlled by a non-genomic estrogen-activated ERα pathway through regulating MIR21 levels^26^.

MIR21 is one of the most frequently identified miRNA species in human bone and changes in its circulating levels are significantly associated with osteoporotic risk however the results are inconsistent as some studies indicated elevated levels of MIR21 in serum and bone tissue of osteoporotic subjects^26–28^, which may suggest elevated MIR21 as a risk of osteoporosis. Other studies reported reduced circulating MIR21 levels in osteoporotic and osteopenia subjects and the decrease in serum MIR21 was significantly associated with increased risk of vertebral fracture and low bone mass^29,30^. The results of our study indicated a positive association between the bone expression and circulating MIR21 levels with age and sBAP in all subjects under the study (male and premenopausal and postmenopausal females) which further confirms the contribution of estrogen hormone. Estrogen deficiency often presents enhanced bone resorption, reduction of bone mineral density, and accelerated osteoporosis occurrence^31^. Many molecular factors are involved in these effects including multiple miRNAs, pro-inflammatory mediators, as well as BMPR2, SMAD1, 5, and 7, TNFSF11, and MMPs which are known to perform important roles in regulating bone metabolism^32^.

Signaling pathways such as Wnt, BMP2, FGF, and IGFBP/IGF have been reported to control the BAP expression. Activation of BMP-BMPR-SMADs signaling and/or FGF-FGFR-MAPK signaling increases BAP expression and osteoblast differentiation^33^.

The circulating level of MIR34a is significantly higher in the postmenopausal females compared with the males while no significant difference was observed between the pre- and post-menopause females. While the bone expression is highest in the premenopausal females compared with males and postmenopausal females. The circulating MIR34a showed no association with age. While bone expression was positively associated with age, MIR34a is suggested to be a potential suppressor of osteoclastogenesis and calcium reabsorption in bones^34^. Overexpression of MIR34a in experimental animals had lower bone resorption and higher bone mass while animals with MIR34a knockout showed opposite results^35^. However, it was reported that MIR34a is a dual-effector miRNA with regulatory effects on osteoblastic proliferation and differentiation as its overexpression suppressed osteoblastic differentiation of human stromal stem cells, and conversely, in vivo, bone formation was induced in the case of MIR34a deficient human stromal stem cells^36^. A previous study reported elevated circulating levels of MIR34a among old adults^37^.

Regarding MIR155, the highest circulating and bone expression levels were observed in the premenopausal females. It was reported that the upregulated miRNA-155 expression could stimulate osteogenic differentiation by directly inhibiting SOCS1 (transcription factor for osteoclast differentiation) gene expression which causes BMP-2-induced osteoblast differentiation^38^. Meanwhile, the downregulation of miRNA-155 increased the SOCS1 protein level resulting in the development of osteoporosis^39^. In addition, the miRNA-155 expression was proved to be negatively correlated with estrogen level^40^. In line with this, our study indicated a positive association between the circulating and bone expression levels of MIR155 and the age in postmenopausal females only. Men in general exhibited a lower perception of fracture risk than females^41^ which coincident in the present study with the tendency of higher bone expression of MIR21 and MIR155 in females compared to male bone.

Since the importance of genes controlling osteogenesis and resorption including BMPR2, SMAD1, 5, and 7, TNFSF11, and MMP9 we assessed their expression in the bone tissues of the studied subjects. The results indicated higher expression levels of BMPR2 and SMAD7 in postmenopausal females than in males and premenopausal females. Also, TNFSF11 bone expression is higher in premenopausal females than males and postmenopausal females, while no significant differences between the studied groups regarding the bone expression of SMAD1 and 7 and MMP9. Since BMPR2, SMAD1/5/8, and TNFSF11 genes give signals that control osteoblast and osteoclast proliferation and differentiation, and MMP9 induces bone resorption, we thought they would be useful to reveal their relationships with the circulating miRs.

The correlation of bone expression of the five studied genes (BMPR2, SMAD1, SMAD5, SMAD7, TNFSF11, and MMP9) with the circulating levels of the three studied three microRNAs (MIR21, MIR34a, and MIR155) worth analyzing to investigate these miRs as a novel indirect estimate of bone quality in both males and pre and postmenopausal women. The circulating MIR21 was positively associated with the bone expression of BMPR2 in all groups, positively associated with SMAD1 and 5, and MMP9 in both males and premenopausal females while negatively associated with SMAD7 in all groups. In line with these data, it was reported that MIR21 targets SMAD7 to promote osteogenic differentiation through the SMAD1/5/8 pathway^42,43^. The literature showed that one of the target genes for MIR21a is SMAD7 which is known for its antagonistic role in TGF-β1 signaling. Certainly, MIR21 promotes BM-MSCs bone formation and this process is regulated, in part, by the SMAD7-SMAD1/ 5/8-RUNX2 pathway^42^.

The circulating MIR155 showed an interesting pattern of correlations as it showed a strong positive association with BMPR2, SMAD1 and 5, and MMP9 in all studied patients. While it is negatively associated with bone expression of TNFSF11 in the postmenopausal females and positively associated with TNFSF11 expression in the premenopausal females. Also, it is negatively associated with SMAD7 in the premenopausal females. In contrast to our results, the previous study reported that MIR155 targeted BMPR2 to inhibit osteogenesis in the C2Cl2 myoblast cell line^44,45^.

The circulating MIR34a showed the least association with bone status as it is negatively associated with TNFSF11 in males and postmenopausal females and positively associated with MMP9 in males and premenopausal females. Also, it is negatively associated with SMAD7 in the premenopausal females. It was reported that MIR34a inhibits bone resorption and osteoclast differentiation by targeting and down-regulating TNFSF11 expression at the posttranscriptional level, through enhanced mRNA degradation and other factors which downregulates the RANK/TNFSF11 signaling pathway^46,47^. On the other hand, elevated levels of MIR34a also promoted osteogenic differentiation of mesenchymal stromal cells which may have implications for osteoporosis^48^.

Our findings provide evidence of the association between the circulating miRNA levels with their bone expression and the expression of genes regulating bone formation and resorption which is of great importance for the application of circulating miRNAs as markers of bone health, particularly in conditions where bone samples cannot be obtained. The clear results of MIR21 and MIR155 in serum and bone tissue and their correlations with bone markers and genes may suggest the circulating levels of these microRNAs as a signature that may provide valuable information of bone health and quality and may provide early markers for the prediction of bone diseases and fracture risk. These positioned us to carry out future studies relating these miRNAs to changes in bone quality in different bone diseases in comparison with the healthy populations which may provide a platform to improve bone quality assessment and may ultimately lead to new methods to enhance bone health.

To our knowledge the present study is the first study to demonstrate the detection of miRNAs 21,155 and 34a expression together and their target genes regulating bone formation and resorption in male and pre- and postmenopausal female bone samples and correlated them with the bone health status however the study has several limitations. A more in-depth study of the relationship between miRNA expression in bone and serum with BMD is required. Also, analysis of miRNA expression in bone samples from our population exhibits low statistical power due to the small sample size and may have been influenced by the prior or current medical conditions of these individuals.

## Conclusion

In conclusion, our study focused on three miRNAs (MIR21, MIR34a, and MIR155) as markers of bone quality in human subjects. The assessment of circulating levels of the selected miRNAs in human serum and bone expression and their correlation.

### Methods

The research was conducted following the World Medical Association’s Code of Ethics (Declaration of Helsinki). The study was conducted under the Declaration of Helsinki, and approved by the Ethics Committee of Medical Research Institute, Alexandria University, Egypt. (Approval serial number: E/C, S/N, R18/2020 in October 2020). Written informed consent was obtained from all participants included in the study.

This prospective study was conducted on 60 patients (30 males and 30 females) admitted to the Department of Neurosurgery, Faculty of Medicine, Alexandria University, Egypt, during the period between October 2020 and September 2021. All patients were subjected to history taking, clinical and neurological examination, and preoperative imaging (X-ray and MRI spine). Postmenopausal women were defined as those over the age of 50 years who have not had menses for the past 5 years. Premenopausal women were those aged 18–45 with regular menstrual cycles. All the participants were presented with either lumbar disc prolapse, lumbar canal stenosis, or lumbar spondylolisthesis. All patients were indicated for surgical interventions for neural decompression +/- fixation. Bone excision was an integral part of these spinal surgeries during laminectomy, hemilaminectomy, or even fenestration. The bone health status of all the participants was monitored preoperatively by assessment of serum bone biomarkers; osteocalcin and bone alkaline phosphatase (sBAP).

Serum samples: Fasting blood samples were collected from participants before the surgery and stored at 4 C. The samples were allowed to clot at 4 ˚C for 30 min, and then centrifuged (2920×g) for 15 min to obtain serum and stored at -80 ˚C until further use.

Bone collection; the amount of bone needed for molecular analysis (about 1 gram) was collected during spinal surgeries that were done during data collection for the research. The already removed bone for the surgical approach was used as a bone sample for the research.

### MicroRNA Selection

We have searched the TarBase v7.0^49^ for the selection of the miRNAs validated as interacting with target genes related to bone metabolism (osteogenesis and clastogenesis) by manual search of individual genes using Ensembl code. Each hsa-miRNA was selected for analyses when the database reported an association (positive result) and the source tissue specified as bone. After this survey, the obtained RNAs list was compared with the reported miRNAs in 20 reviews obtained by comprehensive literature review using keywords; miRNA AND bone-turnover AND gene^13–22,50–59^, any miRNA in the list which was reported in at least in 10 studies was selected. Using this strategy 6 miRNAs were rendered eligible for analyses arranged from the highest to the lowest reported as follows: MIR21, MIR34a, MIR155, MIR124, MIR125, and MIR133. In the present study, we assessed and evaluated the first three miRNAs: MIR21, MIR34a, and MIR155.

### Assessment of serum osteocalcin and bone-specific alkaline phosphatase (sBAP)

The serum levels of osteocalcin and sBAP were assayed using a human-specific ELISA kit (Elabscience, cat. no.E-EL-H1343, and Cusabio, cat. no. CSB-E09033h, respectively) according to the manufacturer’s instructions.

### Molecular analysis of serum samples

#### Isolation of total RNA from serum

We used miRNeasy serum/plasma RNA isolation kit (Cat No. 217184, Qiagen, Inc.) due to its favorable performance in the isolation of total and miRNA suitable for RT-qPCR detection assays^60^ The serum samples were thawed completely on ice and mixed by inversion immediately. One ml of QIAzol solution was added to 200 µL of serum samples, mixed well by vortexing, then incubated at room temperature for 5 min. 200 µL of chloroform was added for phase separation and thoroughly homogenized by vortexing at the maximum setting for 30 s, followed by centrifugation at 12,000×g for 15 min at 4 °C. After centrifugation, the sample was separated into 3 phases: an upper, colorless, aqueous phase containing RNA; a white interphase; and a lower, red, organic phase. The aqueous phase was transferred to a new tube and 1.5 volumes of 100% ethanol were added and mixed thoroughly by pipetting up and down several times. 700 µl of the mixture was added into the RNeasy MinElute spin column in a 2 ml collection tube, then centrifuged at 8000×g for 15 s at room temperature and the flow-through was discarded. This step was repeated for the remaining of the mixture. 700 µl of Buffer RWT was added to the spin column, and centrifuged at 8000×g for 15 s at room temperature and the flow-through was discarded. Then 500 µl of Buffer RPE was added onto the spin column and centrifuged at 8000×g for 15 s at room temperature and the flow-through was discarded. 500 µl of 80% ethanol was added onto the spin column and centrifuged at 8000×g for 2 min at room temperature and the flow-through was discarded. Then the spin column was transferred into a new 2 ml collection tube and centrifuged for 5 min to dry the membrane. Finally, the spin column was transferred into the elution tube and 14 µl RNase-free water was directly added to the center of the spin column membrane and centrifuged for 1 min at full speed to elute the RNA. The quantity and purity of eluted RNA were determined using a NanoDrop ND-1000 spectrophotometer (Isogen Life Science, Temse, Belgium). Following extraction and elution, the RNA samples were immediately frozen at − 80 °C until PCR analysis.

#### Quantification of serum levels of miRNAs using RT-qPCR

We used Applied Biosystems™ TaqMan™ miRNA Assays to quantify mature MIR21, MIR34a, and MIR155 (Cat. no. 4427975, ID: 000397, and 000426 for MIR21 and MIR34a and Cat. no. 4440886, ID 467534_mat for MIR155) kit and using MIR16 (Cat. no. 4427975, ID: 000391) as a reference gene^61^. First, the extracted RNA was reverse transcribed using TaqMan™ MicroRNA Reverse Transcription Kit (Cat. no. 4366596) using Megaplex™ RT Primers, Human Pool A v2.1 (Cat. no. 4399966). The RT reaction mixture was prepared (for each sample) as follows: 0.15 µl100mM dNTPs (with dTTP), 1.0 µl MultiScribe™ RT enzyme, 1.5 µl Reverse Transcription Buffer, 0.19 µl RNase inhibitor, and 4.16 µl Nuclease-free Water. 7 µl of RT mixture was combined with 5 µl of RNA sample (containing 5 ng RNA) and mixed well, then 3 µl of Megaplex™ RT Primers, Human Pool A v2.1 was added. After mixing and centrifugation, the plate was placed in a thermal cycler under the following conditions: 16 °C for 30 min, 42 °C for 30 min, and 85 °C for 5 min. The cDNA was used for PCR detection of the studied miRNAs (each sample was run in three replicates) as follows: The PCR reaction mixture was prepared for each reaction by adding 1.0 µl TaqMan™miRNA Assay solution (TaqMan probe and PCR primer set), 10.0 µl TaqMan™ Universal PCR Master Mix (Cat. no. 4304437), and 7.67 µl RNase–free water. Then 1.33 µl cDNA was added to 18.67 µl PCR reaction mixture., then qPCR was performed under the following conditions: enzyme activation at 95 °C for 10 min, and 40 cycles of denaturation at 95 °C for 15 s and annealing/extension at 60 °C for 1 min. Amplification, data acquisition, and analysis were performed using CFX Maestro Software for Bio-Rad CFX Real-Time PCR Systems.CFX96 (Bio-rad laboratories, California, USA). The quantities of miRNAs in serum were determined using the 2^−ΔCt^ method using MIR16 as a normalizer gene.

### Molecular analysis of bone samples

#### Isolation of total RNA from bone samples

Total RNA was extracted from bone samples using the miRNeasy Mini kit (Cat No. 217004, Qiagen, Inc.) after preparation of the bone samples using RNAlater solution^62^. The fresh bones were harvested and any attached tissues were removed before the addition of RNAlater solution and stored at 4˚C for 24 h then the RNAlater solution was eliminated and the samples were stored at ‑80˚C. Frozen bone samples were crushed using a mortar and pestle in liquid nitrogen as previously described^63^ The total RNA was extracted as described by the manufacturer. Briefly, 50 mg bone powder was transferred into a sterile tube and homogenized in 700 QIAzol Lysis Reagent using a sterile syringe and needle (20 gauge) by passing the lysate 10 times through the needle. The homogenate was stood at room temperature for 5 min., then 140 µl of chloroform was added and shaken vigorously for 15 s and the tube was stood again at room temperature for 2–3 min for phase separation. After centrifugation at 12,000×g for 15 min at 4 °C, the subsequent steps were performed as outlined previously in serum.

#### Quantification of bone levels of miRNAs using RT-qPCR

As previously described in serum, we used the same procedure and kits with one exception of using U6 small nuclear 1; RNU6-1 (Cat. no. 4427975, ID: 000391) as a reference gene^61^ for the quantification of bone levels of MIR21, MIR34a, and MIR155.

#### Quantification of relative expression of bone-related genes in bone tissues using RT-qPCR

The RT-qPCR was applied to determine the relative expression of studied genes using specific primer sets for each gene (Table 3). First, the total RNA isolated from bone tissues was reverse transcribed using QuantiTect Reverse Transcription Kit (Catalog no. 205311, Qiagen, Germany) according to the manufacturer’s instructions. The kit comprises 2 main steps: elimination of genomic DNA and reverse transcription. The genomic DNA elimination reaction was prepared on ice by adding 2 µl gDNA Wipeout Buffer to 2 µl of isolated RNA (containing 0.5 µg), and 10 µl RNase-free water. Incubated at 42 °C for 2 min, then immediately placed in ice. Then 1 µl Quantiscript Reverse Transcriptase, 4 µl Quantiscript RT Buffer, and 1 µl RT Primer Mix were added, then mixed and incubated for 15 min at 42 °C. The reaction was inactivated by incubation for 3 min at 95 °C. The PCR reactions were performed in three replicates for each sample using QuantiTect SYBR Green PCR Kit (Qiagen, Cat. no. 204243) as follows: 12.5 µl QuantiTect SYBR Green PCR Master mix was mixed with 1.5 µl of each primer (Forward and reverse), 2 µl of cDNA, and 9.5 µl RNase-free water in a 96-standard well plate. Then the plate was loaded into the Bio-Rad thermal cycler CFX96 (Bio-rad laboratories, California, USA), and the instrument was programmed as follows: initial denaturation at 95 °C for 10 min, and 45 cycles of denaturation at 95 °C for 5 s, annealing at 55 °C for 15 s, and then extension at 72 °C for 15 s. Amplification, data acquisition, and analysis were performed using CFX Maestro Software for Bio-Rad CFX Real-Time PCR Systems. The relative expression of the studied genes was quantified relative to the expression of 18 S ribosomal RNA N5 (RNA18SN5) as a reference gene in the same sample by calculating and normalizing the threshold cycles (Ct) values of the target to that of RNA18SN5 using 2^−ΔΔCt^ method^64^.

**Table 3:** Primer sets of studied genes.

Gene	Accession No.	primer sequence	
MMP9	NM_004994	**F**	GCCACTACTGTGCCTTTGAGTC
		**R**	CCCTCAGAGAATCGCCAGTACT
TNFSF11 (RANKL)	NM_003701	**F**	GCCTTTCAAGGAGCTGTGCAAAA
		**R**	GAGCAAAAGGCTGAGCTTCAAGC
SMAD9	NM_005903	**F**	CAGGAGTTTGCTCAGCTTCTGG
		**R**	GGTGCTGGTTACATCCTGCCG
SMAD2	NM_005901	**F**	GGGTTTTGAAGCCGTCTATCAG
		**R**	CCAACCACTGTAGAGGTCCATTC
SMAD7	NM_005904	**F**	TGTCCAGATGCTGTGCCTTCCT
		**R**	CTCGTCTTCTCCTCCCAGTATG
RNA18SN5	NR_003286	**F**	ACCCGTTGAACCCCATTCGTGA
		**R**	GCCTCACTAAACCATCCAATCGG

### Statistical analysis

Data were fed to the computer and analyzed using IBM SPSS software package version 20.0. (Armonk, NY: IBM Corp). For continuous data, they were tested for normality by the Shapiro-Wilk test. Quantitative data were expressed as median and range (minimum and maximum) for abnormally distributed variables (Figures), and mean, and standard deviation for normally distributed quantitative variables (Tables). A one-way ANOVA test was used for comparing Male, Premenopausal, and Postmenopausal females and followed by a Post Hoc test (Tukey) for pairwise comparison between each two groups. Multiple stepwise regressions and Pearson’s correlation analysis were used to estimate the associations between microRNA levels, age, osteocalcin, and bone-specific alkaline phosphatase bone parameters. The significance of the obtained results was judged at the 5% level

## Data Availability

The datasets generated or analysed during the current study are not publicly available due to participants privacy but are available from the corresponding author on reasonable request.
